# Encoding of touch location and intensity by neurons of the medicinal leech *Hirudo medicinalis*

**DOI:** 10.1186/1471-2202-13-S1-P103

**Published:** 2012-07-16

**Authors:** Friederice Pirschel, Jutta Kretzberg

**Affiliations:** 1 Computational Neuroscience, Institute of Biology and Environmental Sciences, University of Oldenburg, D-26111 Oldenburg, Germany

## 

Even in small neuronal systems sensory stimuli evoke precise behavioral responses. The leech, possessing one of the smallest neuronal systems with ~10.000 neurons, shows as a response to a touch of the skin a locally limited bend away from this touch [1]. This local bending reflex is known to be initiated by only two mechanosensory cells (P ‘pressure’ cells) with overlapping receptive fields and to depend both on touch location and touch intensity [2]. Behaviorally, the animal displays a surprisingly precise performance, discriminating touch locations which are only 9° of the body circumference apart [3].

Thomson and Kristan investigated in their study [3] the encoding of stimulus location based on two P cells with overlapping receptive fields. They analyzed the relative response features of the neuronal responses, in particular the differences of spike counts and latencies of both cells. Thereby, they found that the latency difference is the most accurate encoder of the touch location [3]: touch locations with a distance of 4° can be discriminated reliably based on this feature, while discrimination based on the spike count difference requires a touch location distance of 13°.

Based on this study, we investigate how neurons of the medicinal leech encode information about the combination of stimulus location and intensity. We have recorded intracellularly from P cells and interneurons of the local bending network, while stimulating the skin mechanically with tactile stimuli of varied intensity and location. The neuronal responses of simultaneously recorded pairs of mechanosensory neurons were analyzed by calculating the difference of latencies and of spike counts of both cells. The discrimination performance was evaluated based on a pairwise classification of location distances and intensity differences.

For the estimation of touch location, we found in agreement with Thomson and Kristan [3] that the latency difference of both P cells leads to a reliable classification of small touch location distances, when touch stimuli of higher intensities (40 to 50 mN) are used. Touch stimuli of lower intensities (10 to 30 mN) require larger distances of touch location for a reliable estimation result. In this case, spike count differences show as good classification results as latency difference.

For the estimation of stimulus intensities spike counts provide significantly better classification results than response latencies. The classification of this stimulus property does not improve when using the difference between the responses of two cells compared to using the spike count of a single P cell.

Preliminary results show that features of graded interneuron responses like the slope, the amplitude and the area under the EPSP depend clearly on the touch intensity and in a more complex way on the touch location.

## Conclusions

1. The touch location is accurately encoded by the latency difference of two P cells when higher stimulus intensities are used.

2. Stimulus intensity can be classified best based on the spike count of single cells.

3. The combined encoding of stimulus location and intensity is more complex for low intensity stimuli. This topic needs further investigation on the level of interneuron responses.
